# Carbon Nanotube/Alumina/Polyethersulfone Hybrid Hollow Fiber Membranes with Enhanced Mechanical and Anti-Fouling Properties

**DOI:** 10.3390/nano5031366

**Published:** 2015-08-20

**Authors:** Yi Feng, Kun Wang, Chris H. J. Davies, Huanting Wang

**Affiliations:** 1 Department of Chemical Engineering, Monash University, Clayton, VIC 3800, Australia; E-Mails: yi.feng@moansh.edu (Y.F.); kun.wang@monash.edu (K.W.); 2 Department of Mechanical and Aerospace Engineering, Monash University, Clayton, VIC 3800, Australia; E-Mail: chris.davies@monash.edu

**Keywords:** carbon nanotube, hollow fiber membrane, mechanical property, water treatment

## Abstract

Carbon nanotubes (CNTs) were incorporated into alumina/polyethersulfone hollow fibre membranes to enhance the mechanical property and the efficiency of water treatment. Results show that the incorporation of CNTs can greatly limit the formation of large surface pores, decrease the void size in support layers and improve the porosity and pore connectivity of alumina/polyethersulfone membranes. As a result of such morphology change and pore size change, both improved flux and rejection were achieved in such CNTs/alumina/polyethersulfone membranes. Moreover, the CNTs/alumina/PES membranes show higher antifouling ability and the flux recoveries after being fouled by bovine serum albumin (BSA) and humic acid were improved by 84.1% and 53.2% compared to the samples without CNT incorporation. Besides the improvement in water treatment performance, the incorporation of CNTs enhanced the tensile properties of inorganic/polymer membranes. Therefore, such CNTs/alumina/PES hollow fiber membranes are very promising candidates for good filter media in industry, considering their high efficiency and high mechanical properties.

## 1. Introduction

As fresh water shortage and water contamination is becoming increasingly prevalent, a significant amount of research interest and focus have been directed towards the applications of membrane separation technology to improve both water quality and treatment efficiency [1,2,3]. Compared to other technologies, membrane technologies have advantages, such as ease of operation, minimal impact on permeate quality, little or no chemicals required, low energy consumption, moderate capital costs, *etc.* [4]. According to the market report investigated via Acmite Market Intelligence, global demand on membranes in water treatment and industrial uses was valued at approximately U.S. $15.6 billion in 2012 and the market is expected to reach U.S. $21.22 billion by 2016 [5].Therefore the development of high-performance membranes in water and waste treatment has been placed under the spotlight of scientific research.

Membranes can be classified based on the type of materials like polymeric, inorganic, and hybrid membranes. Polymeric membranes have advantages including low costs and ease of fabrication, high efficiency for the removal of particles, high flexibility, *etc.* [6,7,8]. However, due to the fact that most polymers have a hydrophobic nature and generally poor mechanical properties, polymeric membranes are liable to be fouled and to be physically damaged, especially hollow fibre membranes [9,10].

Inorganic membranes, on the other hand, have better chemical and thermal stability than the polymeric membranes and higher antifouling property due to the hydrophilic nature of most inorganic materials. Therefore, the combination of inorganic and polymeric material to make hybrid membranes has become a key innovation step allowing researchers to tackle the weaknesses of polymeric membranes.

Previous studies on the inorganic/polymer hybrid membranes for water treatment are mainly focused on the improvement in flux and antifouling properties in comparison with the polymeric membranes [11,12,13,14,15,16,17]. The incorporation of hydrophilic inorganic additives usually facilitates the non-solvent intrusion during the phase inversion process to lead to the formation of more surface pores with larger surface pore size thus resulting in the improvement of flux. Moreover, such hybrid membranes usually have a highly hydrophilic membrane surface due to the surface aggregation of inorganic additives; as a result, improved antifouling property was achieved compared to the pristine membranes [18].

However, despite these improvements mentioned above via the incorporation of inorganic additives into polymeric membranes, the rejection was usually sacrificed as the result of the formation of large surface pores [15,19]. This is even more severe when high-loading micron-size inorganic additives were used. Moreover, due to the stiffness of inorganic materials, elongation and tensile toughness were usually sacrificed after the incorporation of inorganic particles [20,21,22]. Therefore, there is a need to tackle these problems and to further improve the efficiency of current inorganic/polymer membranes to meet the ever-growing residential, environmental, and industrial requirements.

Since they were first found in 1991, carbon nanotubes (CNTs) have attracted wide attention due to the high aspect ratio and the unique high mechanical, optical, and electrical properties [23,24]. So far, CNTs have been incorporated into polymer membranes or dense inorganic membranes, mainly to enhance the mechanical properties with few papers focusing on hybrid membranes [25,26,27,28,29]. As discussed in the introduction, inorganic-polymer membranes combined the advantages of both polymeric and inorganic membranes and become more and more attractive for industrial application. Therefore, how to further improve water treatment efficiency is of significance and worth studying. In this work, we chose CNTs as an additive and incorporated them into alumina/polyethersulfone hollow fibre membranes, aiming to achieve hybrid membranes with high mechanical properties and water treatment efficiency.

## 2. Results and Discussion

### 2.1. Morphology

**Figure 1:**
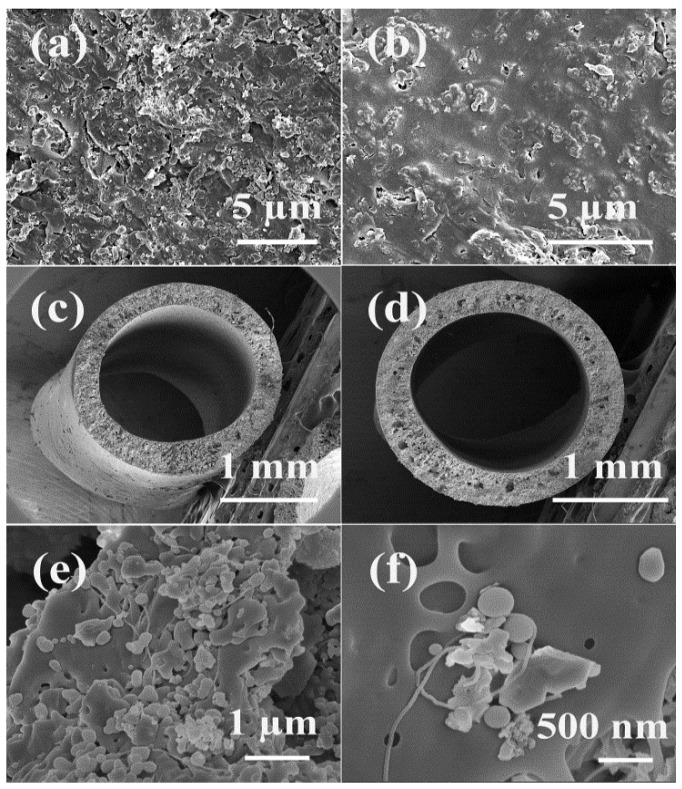
SEM images of membrane surface of samples (**a**) without carbon nanotubes (CNTs) and (**b**) with 0.5 wt % CNTs loading, cross-section of samples with (**c**) 0 wt % and (**d**) 0.5 wt % and (**e**,**f**) CNTs dispersed among alumina and polymer (0.5 wt % CNTs/alumina/PES sample).

Figure 1 shows the SEM images of the morphology of the membrane surface and the cross-section of alumina/polyethersulfone hollow fibre membranes with and without CNTs. For membranes without CNT incorporation, many alumina particles intruded from the membrane surface, which makes the membrane surface rough (Figure 1a). Additionally, many large voids surrounding the intruding particles were observed. In comparison, with the CNTs’ incorporation, the surface seems smoother and the number of large surface pores was greatly reduced (Figure 1b). This might be due to the increased viscosity of the casting solution resulting from the incorporation of CNTs (the viscosity of casting solution with 0. 0.2, 0.5, and 1.0 wt % CNTs loading is 2.32, 2.56, 2.72, and 2.58 Pa s respectively), which would result in the formation of a denser separation layer and the slow migration and out-diffusion of alumina particles from the polymer [30,31]. It should be noted that when the CNT loading further increased to 1.0 wt % the viscosity decreased, which might result from the fact that agglomeration of CNTs might occur and then affect the efficiency of CNTs.

In terms of cross-section, all the samples show similar asymmetric structure (Figure 1c,d). The inner diameter is about 1.5 mm and the outer diameter is about 2.1 mm. Despite the similarity, the porosity and pore size was changed after the incorporation of CNTs, which will be discussed in the next section.

### 2.2. Porosity and Pore Size Distribution

**Figure 2:**
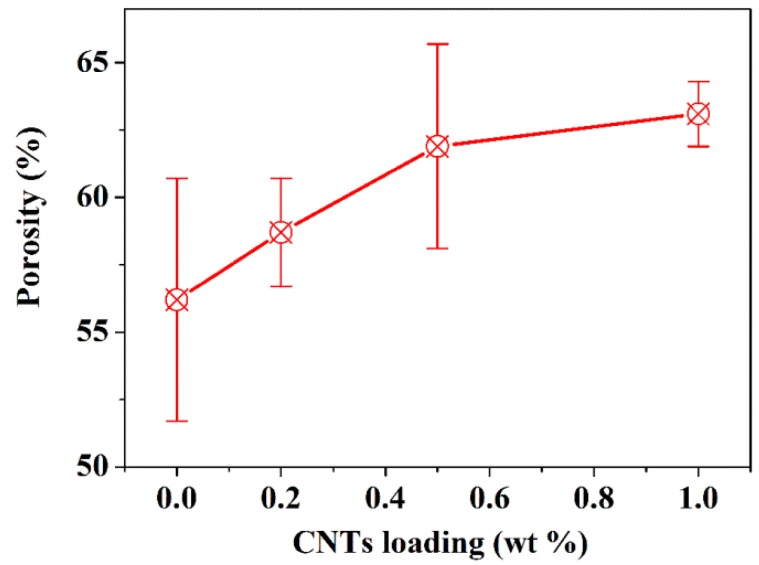
Porosity of alumina/polymer membranes with different CNTs loadings.

**Figure 3:**
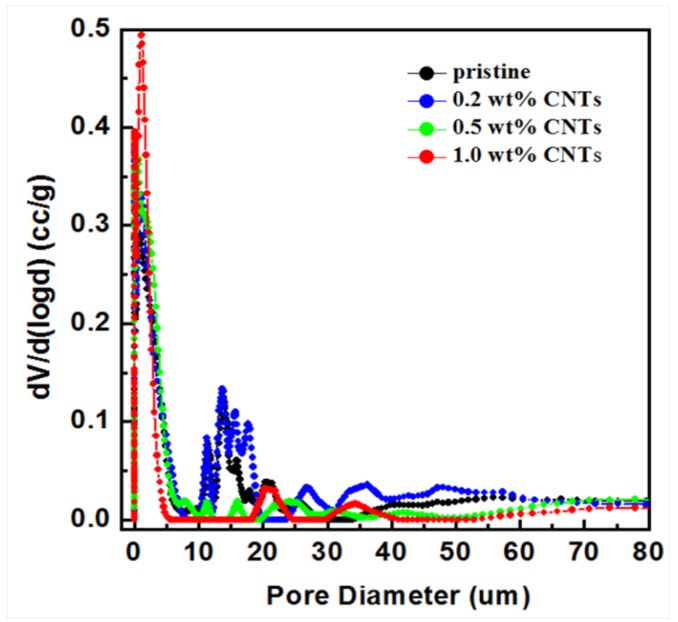
Pore size distribution of hollow fibre membranes analysed by mercury intrusion method.

Table 1 shows the maximum surface pore size of membranes obtained via bubble point test, whereas Figure 2 and Figure 3 give the porosity and pore size distribution. From Table 1, it can be seen that the maximum pore size decreased from 178 nm to 145 nm as the CNTs loading increased from 0.0 wt % to 1.0 wt %. This is consistent with the above hypothesis that the CNTs’ incorporation can reduce the surface pore size.

**Table 1:** Maximum pore size of alumina/polymer membranes with different CNTs loadings.

Sample	Pressure When the First Bubble Appeared (kPa) *	Maximum Pore Size (nm)
Pristine	200	178
0.2 wt % CNTs	205	174
0.5 wt % CNTs	240	148
1.0 wt % CNTs	245	145

Notes: * The pressure showing in this table is the average value of two tests and the standard error is within 10 kPa.

Figure 2 shows the porosity of the membranes with different CNT loadings obtained via the mercury intrusion test. As a general trend, the porosity was increased with the increase of CNT loading and the porosity of 1.0 wt % CNTs/alumina/polymer membrane is approximately 10% higher than the sample without CNTs loading. Due to the high aspect ratio of CNTs, they might act as “bridges” and intertwine among alumina and polymer membranes (Figure 1e); therefore improving the pore interconnectivity. Moreover, the total surface area of membranes (obtained by mercury intrusion) was improved from 13.0 m^2^/g to 17.1 m^2^/g with 1.0 wt % CNTs; thus, better pore connectivity in CNTs/alumina/PES membranes is expected compared to the membranes without CNTs’ incorporation.

Figure 3 shows the pore size distribution of alumina/polymer membranes with different CNT loadings. From Figure 3, it can be seen that for pure alumina/PES hollow fibres and 0.2 wt % CNTs/alumina/PES hollow fibres, many pores with tens of micron were detected. When the CNTs’ loading is higher than 0.2 wt %, the number of pores with the pore size larger than 10 µm were greatly reduced, whereas the number of small pores with pore size about 3–4 µm increased. The decrease of large voids should play a positive role in improving the mechanical properties because of the fact that large voids usually act as crack initiator and lead to continuous cracks under stress.

### 2.3. Mechanical Properties 

**Figure 4:**
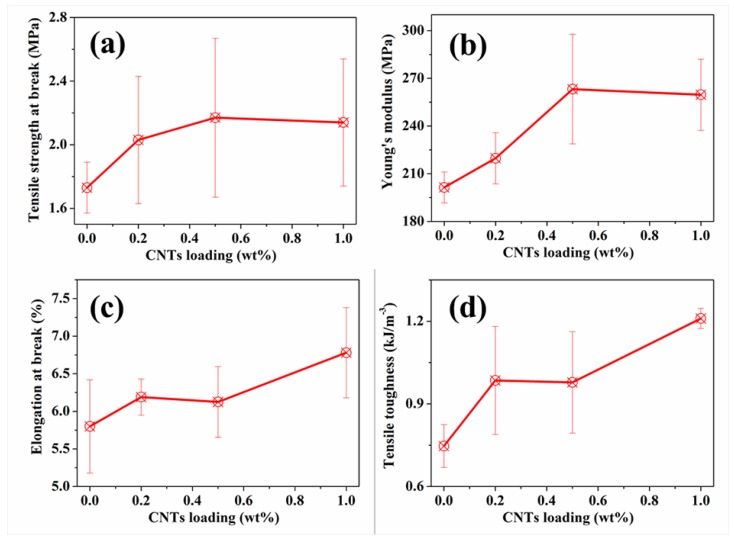
Mechanical properties of alumina/polymer membranes with different CNT loadings: (**a**) Tensile strength at break; (**b**) Young’s modulus; (**c**) Elongation at break and (**d**) Tensile toughness.

Due to their special configuration, hollow fibre membranes are liable to breakage and deformation. Therefore higher tensile strength and Young’s modulus are required. Figure 4 shows the tensile strength, Young’s modulus, elongation at break and the toughness of the pristine membranes and the membranes with CNTs incorporated. It is obvious that the incorporation of CNTs improved the mechanical properties of alumina/polymer hollow fibres. Specifically, the tensile strength was improved by 25.4% with 0.5 wt % CNTs while the Young’s modulus was enhanced by 30.7% with 1.0 wt % CNT loading compared to the pristine membranes (the tensile strength and the Young’s modulus of the pristine membranes are 1.73 MPa and 201.39 MPa, respectively). The well-dispersed CNTs intertwined among the polymer, acting as bridges, which improved the connection among polymers, particles, and the large voids. Additionally, as discussed above, the incorporation of CNTs decreased the pore size of voids in support layers and the large surface pores. These voids usually serve as stress concentrations; therefore, tensile strength and Young’s modulus were improved by the incorporation of CNTs.

**Figure 5:**
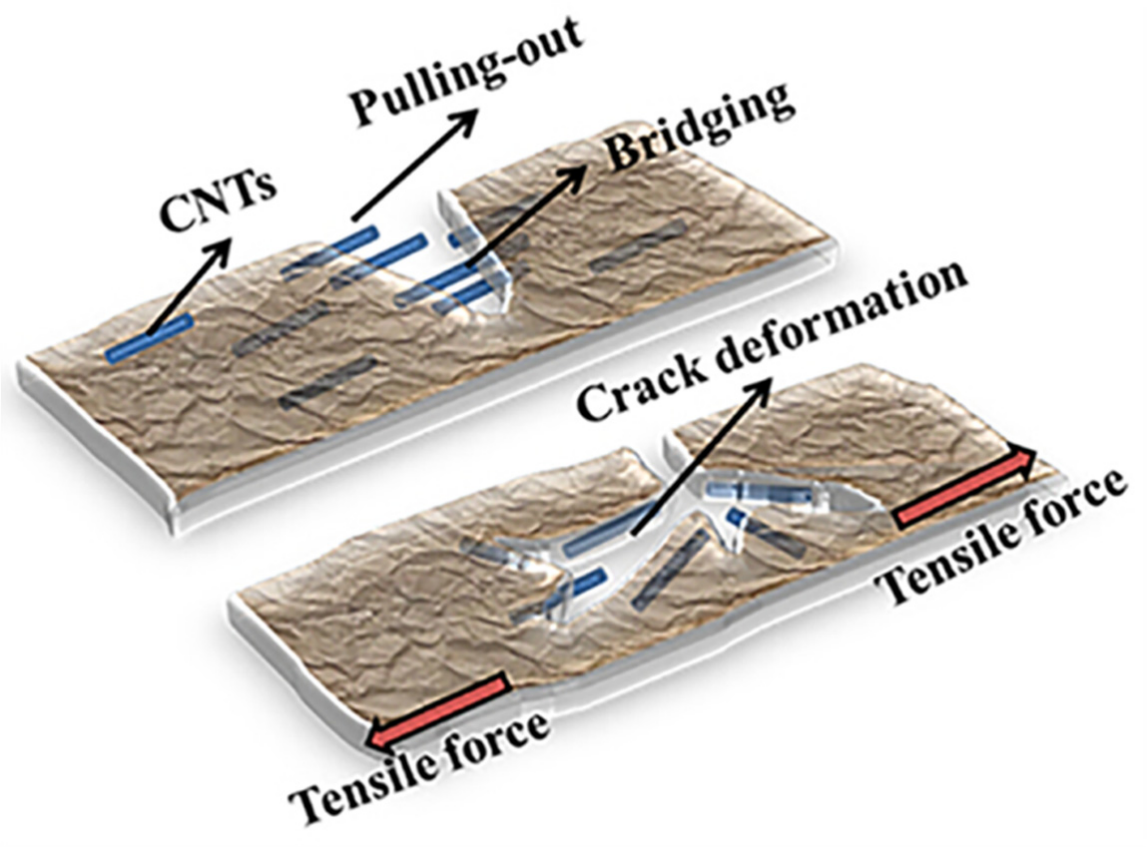
Schematic illustration of CNT-enhanced toughness of alumina/polymer membranes.

In addition to the improved tensile strength and Young’s modulus, the incorporation of CNTs enhanced the toughness (Figure 4d). It is believed that crack deflection and the bridging and pulling-out effects of CNTs are the major contributors to the improvement of CNTs. Due to the intertwined CNTs in the matrices, multi-cracks occurred and no single crack could propagate freely; therefore, more energy was required to break the samples. Moreover, the bridging and pulling out of CNTs from the matrices contributed to the work of fracture since work must be done to pull the fibre ends out of the matrix against the bonding forces, as illustrated in Figure 5. Therefore, toughness was greatly improved due to these effects resulting from the incorporation of CNTs. From Figure 4, 1.0 wt % CNTs loading is considered better than the other loadings in terms of the tensile strength, toughness, and Young’s modulus.

### 2.4. Flux, Rejection and Antifouling Properties

**Figure 6:**
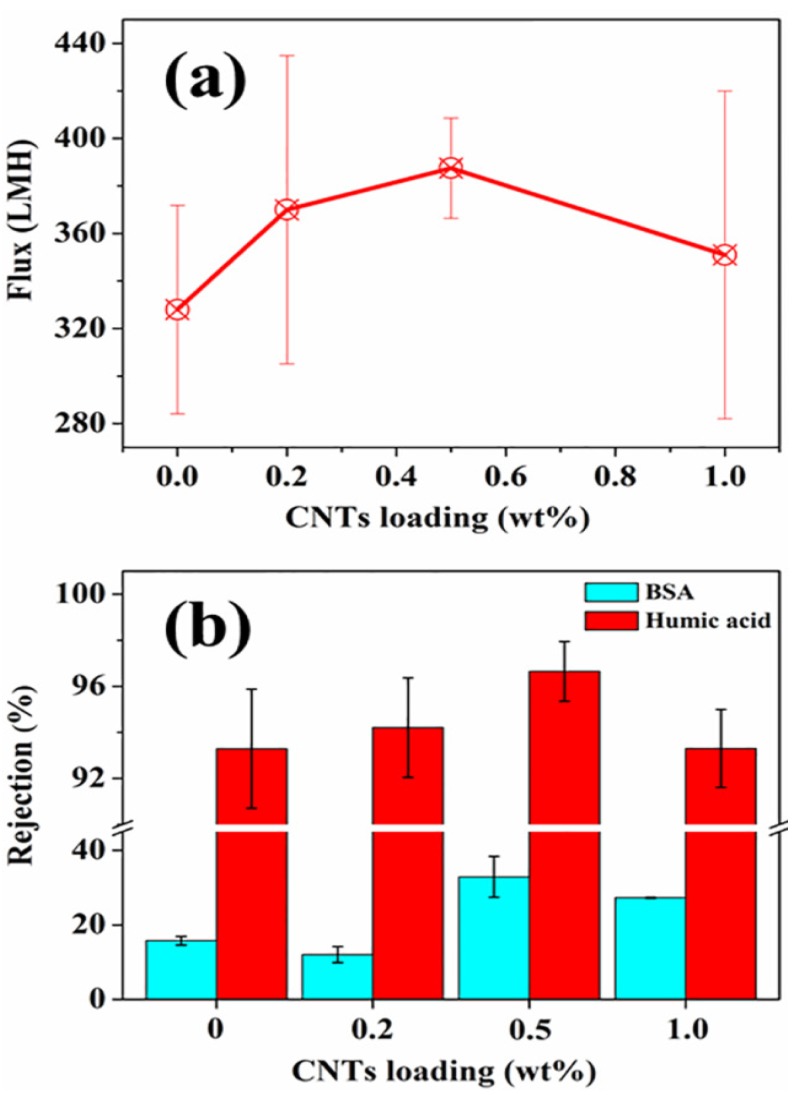
Figure 6**.** (**a**) Flux and (**b**) rejection of alumina/polymer hollow fibre membranes with different CNTs loadings.

Despite the decrease of surface pore size in CNTs/alumina/polymer membranes, the flux was slightly increased in comparison with the alumina/polymer membranes (Figure 6a). As discussed above, the intertwined CNTs inside polymer improved the pore connectivity and the total surface area; therefore, less resistance of water flow was expected in CNTs/alumina/polymer membranes, which might be contributed to the improvement in flux.

In terms of the rejection, due to the decrease of maximum surface pore size and average surface pore size via the incorporation of CNTs, the CNTs/alumina/polymer membranes show higher rejection for BSA and humic acid and the rejection ratios peaked with 0.5 wt % CNTs loading (Figure 6b). However, due to the fact that the maximum surface pore size of our membranes is in the range of 140 nm to 180 nm (Table 1), all the membranes show better rejection for humic acid (>90%), whereas they have poor rejection for BSA (<40%). Compared to other membranes reported in the literature, despite the fact that the maximum pore size of our CNTs/alumina/polymer membranes is in the range of microfiltration, the rejection for humic acid of our membranes is comparable to ultrafiltration membranes reported but the water flux is 2–3 times as high as those ultrafiltration hollow fibre membranes (the flux of reported ultrafiltration membranes is normally less than 150 LMH with the humic acid rejection higher than 95%) [32,33,34]. For microfiltration membranes reported in other studies for humic acid removal, due to the fact that the pore size is usually larger than 0.2 µm, the rejection for humic acid of those membranes is lower than the CNTs/alumina/polymer membranes in this study [35].Therefore, these inorganic/polymer membranes are good filter media for the removal of humic acid from water.

**Figure 7:**
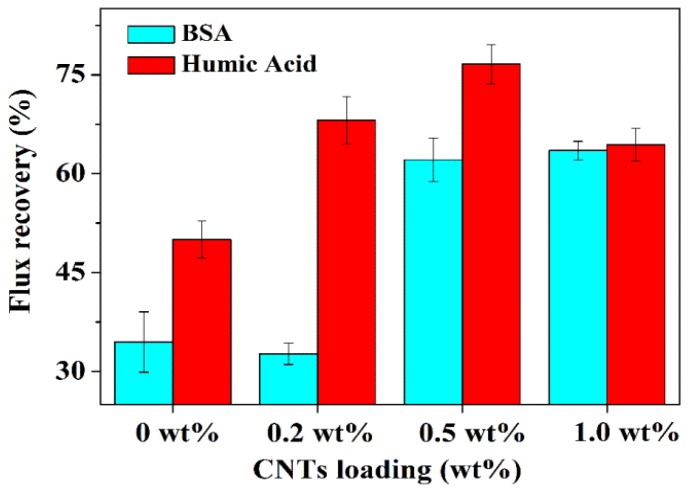
Flux recovery of alumina/polymer hollow fibre membranes with different CNTs loadings.

In addition to the flux and rejection, the antifouling property is another important consideration during the operation. The incorporation of hydrophilic alumina particles can improve the hydrophilicity of membrane surface via the surface aggregation of particles on the interface of polymer and nonsolvent; thus, higher antifouling property would be expected. However, due to the random detachment of alumina from the polymer, a rough surface was obtained in alumina/polymer membranes, as shown in Figure 1. This would greatly limit the antifouling property of membranes. For example, the flux recoveries of alumina/polymer membranes in this study after being fouled by BSA and humic acid are only 34.5% and 50%, respectively (Figure 7).

In comparison, for CNTs/alumina/polymer membranes, the flux recovery of BSA was improved by 84.1% with 1.0 wt % CNT loading, whereas the flux recovery of humic acid was enhanced by 53.2% with 0.5 wt % CNT loading in comparison with the samples without the incorporation of CNTs. As discussed above, the incorporation of flexible CNTs increased the viscosity of casting solution and thus slowed down the migration of alumina particles during the phase inversion process; as a result, more alumina particles might be kept in the water/film interface without intruding from the polymer and smoother surface was formed (Figure 1b). Moreover, because of the above-mentioned effects, a more hydrophilic membrane surface was observed with the incorporation of CNTs (The contact angle of the 0.0 wt %, 0.2 wt %, 0.5 wt % and 1.0 wt % CNTs/alumina/polymer membranes are 45°, 36°, 30° and 35° respectively). Therefore, all these effects resulting from the incorporation of CNTs attributed to the improved flux recovery and antifouling property.

Despite the improvement in antifouling property of CNTs/alumina/PES membranes for both BSA and humic acid compared to membranes without CNTs, such membranes show higher antifouling property for humic acid than BSA, as shown in Figure 7. This might be due to the fact that BSA has smaller size and more BSA would pass the skin layer and cause more severe internal fouling. Therefore, lower flux recovery was obtained in the case of BSA than humic acid.

## 3. Experimental Section

### 3.1. Materials

The chemicals were used as received. They include poly (ether sulfone) (PES, Ultrason E6020P, 51 kDa, BASF, Ludwigshafen, Germany), 1-methyl-2-pyrrolidone (NMP) (anhydrous, purity ≥ 99%, Sigma-Aldrich, Castle Hill, Australia), alumina powder (*d*_50_ = 1.2 μm, PP 5010, Shell-lap Supplies Pty Ltd, Australia), carbon nanotubes (multi-walled, outside diameter is 10–20 nm, inside diameter is 3–5 nm, the length is 5–30 µm, SkySpring Nanomaterials, Houston, TX, USA), humic acid (technical grade, Sigma-Aldrich, Castle Hill, Australia), bovine serum albumin (BSA) (agarose gel electrophoresis, Sigma-Aldrich, Castle Hill, Australia).

### 3.2. Sample Preparation

The inorganic/polymer and the CNTs/inorganic/polymer hollow fibre membranes were prepared via the nonsolvent induced phase inversion method at room temperature [36]. Specifically, CNTs were first dispersed into NMP via ultrasonification, followed by the addition of PES polymer and alumina particles. The ratio of PES: NMP: Al_2_O_3_ powders is 7:46:47 (wt %) (3.5 g PES, 23 g NMP and 23.5 g Al_2_O_3_ powders). The CNTs loading is varied as 0.2 wt % (47 mg), 0.5 wt % (118 mg) and 1.0 wt % (235 mg) based on the weight of alumina. The obtained CNTs/alumina/PES suspensions were then ball-milled at a speed of 20 rpm for at least 2 days to obtain the homogeneous mixture followed by degassing overnight. The achieved suspensions were then extruded through a tube-in-orifice spinneret (the outer diameter is 2.6 mm and the inner diameter is 1.6 mm) using pressurized nitrogen gas. Double de-ionized (DDI) water was used as inner and outer coagulant and the air gap was set as 4 cm. The obtained hollow fibre precursors were maintained in outer coagulant until use.

### 3.3. Characterizations

#### 3.3.1. Morphology and Surface Hydrophilicity

The cross-sections of membranes were prepared via fracturing membranes in liquid nitrogen and then examined using scanning electron microscopy (Nova Nano SEM, FEI Company, Hillsboro, OR, USA); the top surface of membranes was characterized using scanning electron microscopy (Magellan SEM, FEI Company, Hillsboro, OR, USA). All the SEM work was performed at an accelerating voltage of 5 kV with the secondary electron (SE) detector and all samples were coated with Pt. The total porosity, total surface area, and pore size distribution of the samples were determined via mercury intrusion (Auto pore III, Micromeritics, Norcross, Switzerland). The viscosity of casting solution was measured via rheometer (HAAKE MARS Rheometer, Thermo Electron Corporation, Waltham, MA, USA). The hydrophilicity of hollow fibre surface was measured via the captive bubble method and the contact angle was recorded and measured via the video-based optical contact angle measuring instrument (OCA-15EC, Dataphysics, Filderstadt, Germany).

#### 3.3.2. Mechanical Properties

The mechanical properties of hollow fibres were measured using mini-instron (Micro Tester 5848, 100 N load cell, Instron Calibration Laboratory, Buckinghamshire, UK). Before the tensile test, Torr Seal (low vapour pressure resin, Varian, Jefferson Hills, PA, USA) was used to seal both ends of hollow fibres to keep the configuration of fibres at both ends and to ensure the crack does not occur at the fixing points. Tensile strength and elongation were measured with a 30 mm gauge length and a constant elongation velocity of 0.5 mm/min. The tensile Young’s modulus was calculated based on the stress-strain curve with the range of 0.5%–1.0% tensile strain. The toughness was calculated based on the area under the stress-strain curve. For every sample, at least five specimens were tested.

#### 3.3.3. Flux, Rejection and Antifouling

The filtration test was carried out in HP4750 cell (Sterlitech, Kent, WA, USA) with compressed nitrogen gas to control the feed pressure. [37] To fix the fibre membrane, the non-porous stainless steel supporting disc with a circular hole in the centre was used. The disc has a diameter of 50 mm and a thickness of 2 mm meanwhile the hole in its centre has a diameter of 2 mm. The hollow fibre membrane was placed perpendicularly to the supporting disc in the hole. An epoxy resin sealant (Varian Vacuum Technologies, Jefferson Hills, PA, USA) was used to seal the top end of the membrane and the space between the membrane and the supporting disc.The permeate water was accumulated on a beaker sitting on top of an electronic balance and its mass change was automatically recorded. During the flux test, 150 kPa was used to precompact the membrane and the flux was tested and recorded at the pressure of 100 kPa (denoted as *J_w_*_1_).

For the rejection and antifouling test, 1.0 mg/mL BSA/PBS buffer (pH = 7.4) and 10 ppm humic acid were used as foulants respectively. The rejection ratio (R) is calculated using the following equation:

(1) R(%)=(1−CpCf)∗100

where *C_p_* and *C_f_* were the foulant concentrations of permeate and feed solutions, respectively. The concentrations of BSA solution were determined based on the absorbance at 280 nm and the concentrations of humic acid were determined at 308 nm using a UV spectroscope (UV mini-1240 spectrophotometer, Shimadzu, Kyoto, Japan).

After fouling, the membranes were cleaned with double deionized (DDI) water; the cell was then emptied and the pure water flux was measured again (now denoted as *J_w_*_2_). To evaluate the antifouling property of the membranes, the flux recovery ratio (FRR) is calculated using the following equation:

(2) FRR(%)=(Jw2Jw1)∗100

## 4. Conclusions

The incorporation of CNTs can greatly limit the formation of large surface pores and decrease the void size in support layers, yet improve the porosity and pore connectivity of alumina/polymer hybrid membranes via increasing the viscosity of casting solution and slowing the migration of alumina particles during the phase inversion process. As a result of these morphology changes, both improved flux and rejection were achieved in CNTs/alumina/polymer hollow fibre membranes compared to the samples without CNT incorporation. Moreover, due to the smoother yet more hydrophilic membrane surfaces, CNTs/alumina/polymer membranes show higher antifouling property. In terms of the mechanical properties, all the tensile properties (strength, Young’s modulus, elongation, and toughness) were enhanced after the incorporation of CNTs. Taking the mechanical and filtration performance into consideration, 0.5 wt % CNT loading is optimal in this study and such CNTs/alumina/polymer hollow fibre membranes are very promising to be used as filter media in practical industrial applications such as the removal of humic acid.
